# Immune checkpoint inhibitors use and effects on prognosis of COVID-19 infection: a systematic review and meta-analysis

**DOI:** 10.2217/imt-2021-0007

**Published:** 2021-08-25

**Authors:** Wenwei Qian, Ying Ye, Lugen Zuo, Ting Song, Qing Xu, Yinghong Wang, Jun Qian, Yun Tian

**Affiliations:** ^1^Department of General Surgery, Jinling Hospital, Medical School of Southeast University, No. 305 East Zhongshan Road, Nanjing, PR China; ^2^Emergency Center, Affiliated Hospital of Xuzhou Medical College. 99 Huaihaixi Road, Xuzhou, Jiangsu, 221002, China; ^3^Department of Gastrointestinal Surgery, First Affiliated Hospital of Bengbu Medical College, Bengbu, Anhui, 233004, China; ^4^Department of Infectious Diseases, The Sixth People’s Hospital of Qingdao, No. 9 Fushun Road, Qingdao, Shandong, 266033, China; ^5^Department of Oncology, Tongji University Cancer Center, The Shanghai Tenth People’s Hospital, Tongji University, Shanghai, China; ^6^Department of Gastroenterology, Hepatology & Nutrition, The University of MD Anderson Cancer Center, Houston, TX 77030, USA; ^7^Department of Oncology, Jiangsu Province Hospital of Chinese Medicine, Affiliated Hospital of Nanjing University of Chinese Medicine, Nanjing, 210029, China

**Keywords:** COVID-19, hospitalization, immune checkpoint inhibitor, meta-analysis, mortality, prognosis, severe disease

## Abstract

**Aim:** We aimed to quantify the effects of immune checkpoint inhibitors (ICIs) on the prognosis of COVID-19. **Materials & methods:** A meta-analysis was conducted and the hospitalization, severe disease and mortality rates were assessed. Thirteen studies comprising of 4614 cancer patients with COVID-19 were included. **Results:** When compared with cancer patients without prior ICI exposure, patients with prior ICI treatment exhibited a higher rate of hospitalization (odds ratio [OR] 2.0, 95% CI 1.19–3.38, p = 0.01). However, the OR of severe disease and mortality in ICI exposed cases was similar to non-ICI exposed patients (OR 1.55, 95% CI 0.69–3.51, p = 0.29; OR 1.12, 95% CI 0.85–1.48, p = 0.42, respectively). **Conclusion:** It is uncertain whether prior exposure to ICIs increases the risk of severe disease and death, however the observed OR suggest a higher rate of hospitalization.

## Lay abstract

COVID-19 is an infectious disease caused by a virus which affected people worldwide in 2020. It mainly attacks the lungs and causes symptoms such as, fever, dry cough and fatigue. However, there is currently are no definite therapies for its treatment. Cancer patients are more vulnerable due to both the tumor itself and the anticancer treatment. At the same time, they are at higher risk of COVID-19 exposure due to the need for regular treatment and testing in hospitals. In this systematic review and meta-analysis we enrolled 13 studies. Firstly, we analyzed the rate of hospitalization, severe disease and death. Additionally, we studied the impact of immune checkpoint inhibitors on the outcome of cancer patients infected with COVID-19. Finally, our discussion focuses on what we can learn from the pandemic to provide guidance for clinical practice.

COVID-19 has already spread quickly on a global scale, evolving into a pandemic and threatening global health [1–3]. The rapid rise of the disease, and the resultant hospitalizations and deaths have strained public health systems [4]. It mainly attacks the lungs and causes related symptoms, including fever, dry cough and fatigue etc., [5]. In addition to lung injury, COVID-19 is associated with hepatitis, gastrointestinal symptoms (such as diarrhea) and damage to other organs [6]. However, there currently are no effective therapies for its treatment. Cancer patients are often at higher risk of COVID-19 exposure due to the need for regular treatment and testing in hospitals [7]. To make matters worse, cancer patients are more vulnerable due to both the tumor itself and the anticancer treatment [8,9]. It remains to be seen whether the application of anticancer drugs results in differential prognoses for patients infected with COVID-19 [10]. It is critically important for clinicians to identify risk factors associated with severity and mortality and take appropriate interventions.

Currently, immunotherapy has raised major concerns amid different therapeutic strategies in cancer treatment, due to its intrinsic and extensive influence on the immune system [11]. Immunotherapy primarily consists of several immune checkpoint inhibitors (ICIs), including inhibitors targeting the CTLA-4, PD-1 and PD-L1. Anti-CTLA-4 and anti-PD-1/-PD-L1 antibodies reactivate cytotoxic CD8^+^ T cells for antitumor activity through targeting T-cell exhaustion pathways [12]. In patients with malignancy, ICIs sometimes induces adverse events, including liver injury, pneumonia and colitis [13]. Given the convergence of the downstream effects on innate immunity and organ damage caused by both ICIs and COVID-19 infections, we investigated whether patients present worse prognosis due to prior exposure to ICIs.

Aeppli *et al.* performed an online survey among clinicians involved in the treatment of renal cell carcinoma [14]. The results reflected that over 80% of experts choose pilimumab/nivolumab outside the pandemic, however this figure has fallen by half during the COVID-19 pandemic. Given that ICI therapy represents an important treatment choice for some patients, whether this has an impact on the prognosis of COVID-19 infection in cancer patients should be elucidated. However, whether COVID-19 patients receiving ICI therapy are prone to poorer prognosis remains unknown. In light of this, this systematic review and meta-analysis aimed to assess the safety of ICI application in COVID-19 patients and to make reasonable recommendations by reviewing available publications.

## Materials & methods

### Search strategy

We searched the PubMed, Embase, and Web of Science databases, limiting our search to papers written in English from the inception of each database until 4 January 2021. The search terms were as follows: ‘severe acute respiratory syndrome coronavirus 2’ or 'SARS-CoV-2’ or '2019-nCoV' or 'COVID-19' and ‘cancer’ or ‘malignancy’ or 'tumor' and ‘immune checkpoint inhibitors’ or 'PD-1/PD-L1' or 'CTLA-4' or 'immunotherapy'. Articles were also retrieved by screening the reference lists of included studies and from related review papers. One reviewer (Y Tian) with experience in database searches designed the search, and two reviewers (W Qian and Y Tian) independently screened the titles, abstracts and full text according to these eligibility criteria, assessing the eligibility of publications.

### Inclusion criteria

We included randomized controlled trials, observational studies and case series that reported ICI use in cancer patients and their prognosis in the context of COVID-19. Exclusion criteria were as follows: the same patients enrolled in different studies, studies such as clinical reviews, summaries of meetings, or erratum that did not report original data; and studies containing less than four ICI users. When data was inadequate in some studies, attempts were made to contact the investigators for the missing data.

### Data extraction & definitions

Two researchers (W Qian and Y Tian) independently extracted data from the included studies in a double-blind manner. Any disagreements were resolved by a third investigator (L Zuo) or by consensus. The following variables was extracted: name of first author, country, date of COVID-19 diagnosis, study type, age, gender, total number of patients, number of patients receiving ICI, treatment interval before diagnosis of COVID-19 and outcome of infection, such as hospitalization and/or severity and/or mortality (Table 1). Severe disease was defined according to the original studies, primarily based on the symptoms present during treatment – for example, admission to the intensive care unit, development of severe or critical symptoms and utilization of invasive mechanical ventilation [8]. To ensure high-quality evidence, this study was performed in accordance with the Preferred Reporting Items of Systematic Reviews and Meta-analysis statement.

**Table 1.** Main characteristics of the included studies in meta-analysis.

Study	Year	Country	Sample (n)	Male	ICI users	Age	Study type	Outcome	Treatment interval before diagnosis	NOS
Albiges *et al.*	2020	France	178	76	19	61 (IQR 52–71)	Retrospective	Severity and mortality	3 months	8
Bersanelli *et al.*	2020	Italy	9	8	9	75 (range 50–82)	Prospective	Mortality	21 weeks	6
Dai *et al.*	2020	China	641	302	6	Male median: 64 Female median: 63.5	Prospective	Mortality and severity	40 days	8
Lara *et al.*	2020	USA	121	NR	8	64 (IQR 51–73)	Retrospective	Hospitalization, severity and mortality	NR	5
Lee *et al.*	2020	UK	800	449	44	69 (range 59–76)	Prospective	Mortality	4 weeks	8
Lievre *et al.*	2020	France	1289	795	110	67 (range 19–100)	Prospective	Mortality	3 months	9
Luo *et al.*	2020	USA	69	33	40	69 (range 31–91)	Retrospective	Hospitalization, severity and mortality	6 weeks (n = 20); 6 months (n = 30); ongoing (n = 40).	6
Moritz *et al.*	2020	Germany	13	7	13	65 (range 26–88)	Retrospective	Severity and mortality	51 days	6
Noguera *et al.*	2020	Spain	166	96	58	63 (range 33–86)	Retrospective	Hospitalization	2 months	7
Pinato *et al.*	2020	UK, Italy, Spain and Germany	890	503	56	68 (range 21–99)	Retrospective	Mortality	4 weeks	6
Robilotti *et al.*	2020	USA	423	212	31	416 pts >18 (98%) 234 pts >60 (56%)	Retrospective	Hospitalization and Severity	90 days	5
Szabados *et al.*	2020	UK	4	4	4	67 (range 52–72)	Prospective	Hospitalization, severity and mortality	90 days	5
Wu *et al.*	2020	China	11	8	11	66 (range 29–73)	Retrospective	Severity and mortality	50 days	5

ICI: Immune checkpoint inhibitor; IQR: Interquartile range; NR: Not reported; NOS: Newcastle–Ottawa Scale; pts: Patients.

### Quality assessment

The Newcastle–Ottawa Scale (NOS) was used for observational studies to evaluate the methodological quality of the original study (Table 1) [15]. The NOS consists of three parts: patient selection, study comparability and outcome assessment and produces scores ranging from 0 to 9. Studies with NOS scores of >7 were regarded as high quality. The risk of bias was independently assessed by two authors (W Qian and Y Tian).

### Data synthesis & statistical analysis

All statistical analyses in this study were performed using R (version 4.0.2). Odds ratios (OR) were used to describe the ratio of the probability of events occurring in cancer patients treated with different therapies. The q-test was used to calculate heterogeneity among the included studies and I^2^ test was used to describe the percentage of variation across studies that is due to heterogeneity. p < 0.05 or I^2^ > 50% indicated substantial heterogeneity across the articles [16], and a random effects model was used [17]. Otherwise, a fixed-effects model was used. Publication bias was assessed using the Begg funnel plot and the Egger test linear regression test (where at least five studies were available). A p < 0.05 was considered statistically significant.

## Results

### Search results

**Figure 1.**
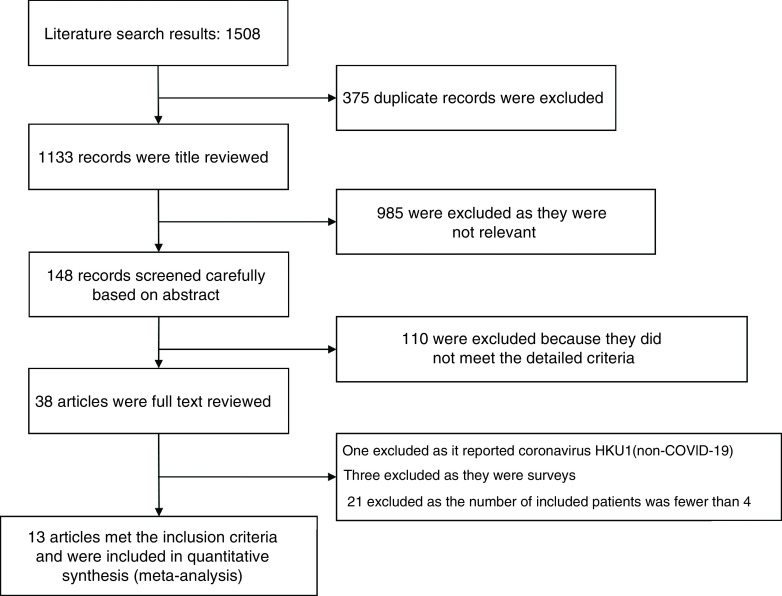
Flow chart for study selection.

The search strategy identified 1508 articles (Figure 1). Among these studies, 375 were duplicates. After screening the title and abstract, 1085 were excluded, and the full text of the remaining 38 articles was reviewed. Among these, one study about coronavirus, three surveys and 21 researches that included less than four patients were excluded after full text review. Finally, 13 studies reported ICI use in cancer patients and prognosis of COVID-19 infection [8,18–29]. The 13 articles consisted of ten cohort studies and three case series and were included for the meta-analysis.

### Patient characteristics

Finally, 13 relevant studies were enrolled, including eight retrospective studies and five prospective studies, comprising more than 4600 cancer patients infected by COVID-19. Detailed patient characteristics of the included studies are shown in Table 1. The studies were from eight countries, including China (n = 2), Germany (n = 2), Italy (n = 2), France (n = 2), Spain (n = 2), the UK (n = 3) and the USA (n = 3). These studies included more than four ICI users, and the median age of study participants was 61–67 years old. Of these 13 studies, clinical outcomes were defined as hospitalization in five studies, severity in eight studies and mortality in 11 studies (Table 1). However, there was nonuniformity in the criterion of the time interval from last dose to COVID-19 diagnosis (Table 1) [8,18,19,21,23–25]. Results of the quality assessment of the included studies assessed by NOS scores are presented in Table 1.

### ICI use & risk of hospitalization in COVID-19 patients

**Figure 2.**
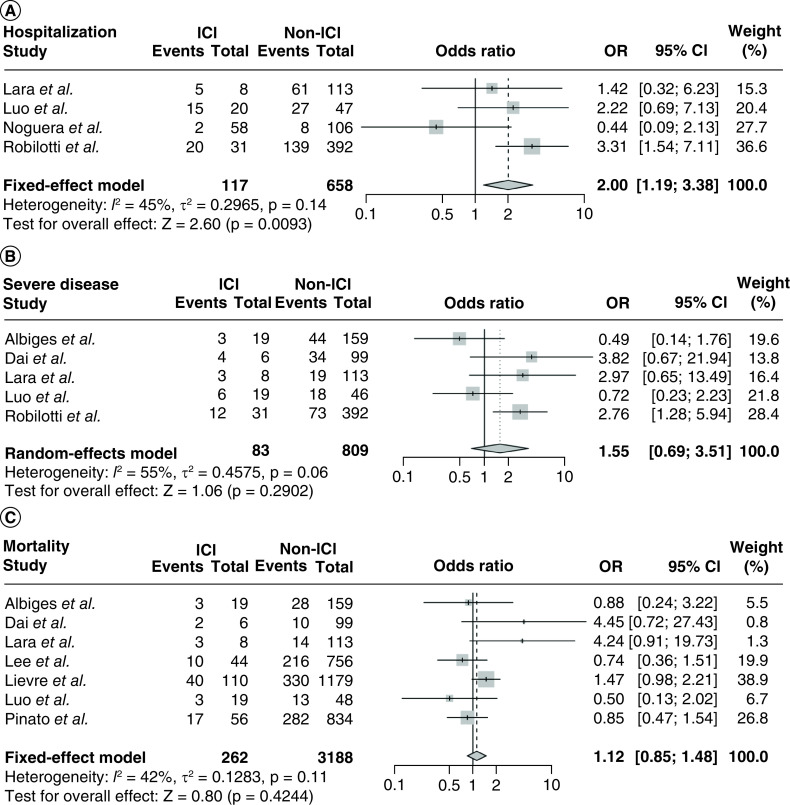
The pooled prognosis of COVID-19 infections compared between patients with prior immune checkpoint inhibitor treatment and those without. ICI: Immune checkpoint inhibitor; OR: Odds ratio.

We combined five studies [20,23,25,27,28] reporting the hospitalization of COVID-19 infection in patients on ICI treatment, and a random effects model was used since the heterogeneity test suggested obvious heterogeneity (I^2^ = 87%, p < 0.01). The pooled estimate of the rate of hospitalization was 0.45 (95% CI 0.15–0.78; Table 2). Four of the five studies [20,23,25,27] contained hospitalization information of patients without ICI exposure. The proportion of hospitalization was markedly increased in patients treated with ICI therapy compared with those without ICI treatment (OR 2.00 [95% CI 1.19–3.38], p = 0.01; I^2^ = 45%; Figure 2A).

**Table 2.** The results of the meta-analysis.

	Studies (n)	OR (95% CI)	p-value	Heterogeneity		Model used	Begg’s test	Egger’s test
				I^2^	p-value			
**Single proportions**								
– Hospitalization	5	0.45 (0.15–0.78)	NA	87%	<0.01	Random	0.33	0.45
– Severe disease	8	0.34 (0.26–0.44)	NA	35%	0.13	Fixed	0.62	0.88
– Mortality	11	0.26 (0.17–0.38)	NA	64%	0.20	Random	0.43	0.36
**Binary outcome**								
Hospitalization								
– ICI vs non-ICI	4	2.00 (1.19–3.38)	<0.01	45%	0.14	Fixed	NA	NA
Severe disease								
– ICI vs non-ICI	5	1.55 (0.69–3.51)	0.29	55%	0.06	Random	1.00	0.79
**Mortality**								
– ICI vs Non-ICI	7	1.12 (0.47–1.54)	0.42	42%	0.11	Fixed	0.65	0.85
– ICI vs chemotherapy	6	1.09 (0.54–1.97)	0.56	0%	0.46	Fixed	0.85	0.73
– ICI vs hormone therapy	5	1.45 (0.70–2.97)	0.32	53%	0.08	Random	1.00	0.80
– ICI vs radiotherapy	4	1.13 (0.74–1.74)	0.57	26%	0.26	Fixed	NA	NA
– ICI vs surgery	4	1.69 (0.95–2.98)	0.57	0%	0.64	Fixed	NA	NA
– ICI vs targeted therapy	6	2.13 (1.44–3.14)	<0.01	15%	0.32	Fixed	0.85	0.65

ICI: Immune checkpoint inhibitor; NA: Not available; OR: Odds ratio.

### ICI use & influence on COVID-19 severity

Eight studies [8,18,20,23,24,27–29] that included 111 COVID-19 cases with ICI exposure reported on COVID-19 severity in relation to ICI exposure. The combined proportion of severe disease was 0.34 (95% CI 0.26–0.44, I^2^ = 35%; Table 2). Out of these eight studies, five studies [8,18,20,23,27] included 83 COVID-19 cases with ICI exposure and 809 COVID-19 cases unexposed to ICI. A random-effects model was used (I^2^ = 55%; p = 0.06) and the pooled OR of COVID-19 severity was 1.55 (95% CI, 0.69–3.51, p = 0.29; Figure 2B).

### ICI use & risk of mortality in COVID-19 patients

The overall analysis included 11 studies [8,18–24,26,28,29]. Together, 299 COVID-19 cases with ICI exposure and 3188 COVID-19 cases without ICI exposure were included. The pooled proportion of mortality in COVID-19 patients with ICI exposure was 0.26 (95% CI, 0.17–0.38; Table 2). Next, the risk associated with ICI use and mortality was assessed. Overall, the OR of mortality in ICI-exposed cases was similar to non-ICI exposed COVID-19 patients (OR 1.12, 95% CI 0.85–1.48, p = 0.42; Figure 2C). Moderate heterogeneity was observed among the studies (I^2^ = 42%, p = 0.11).

**Figure 3.**
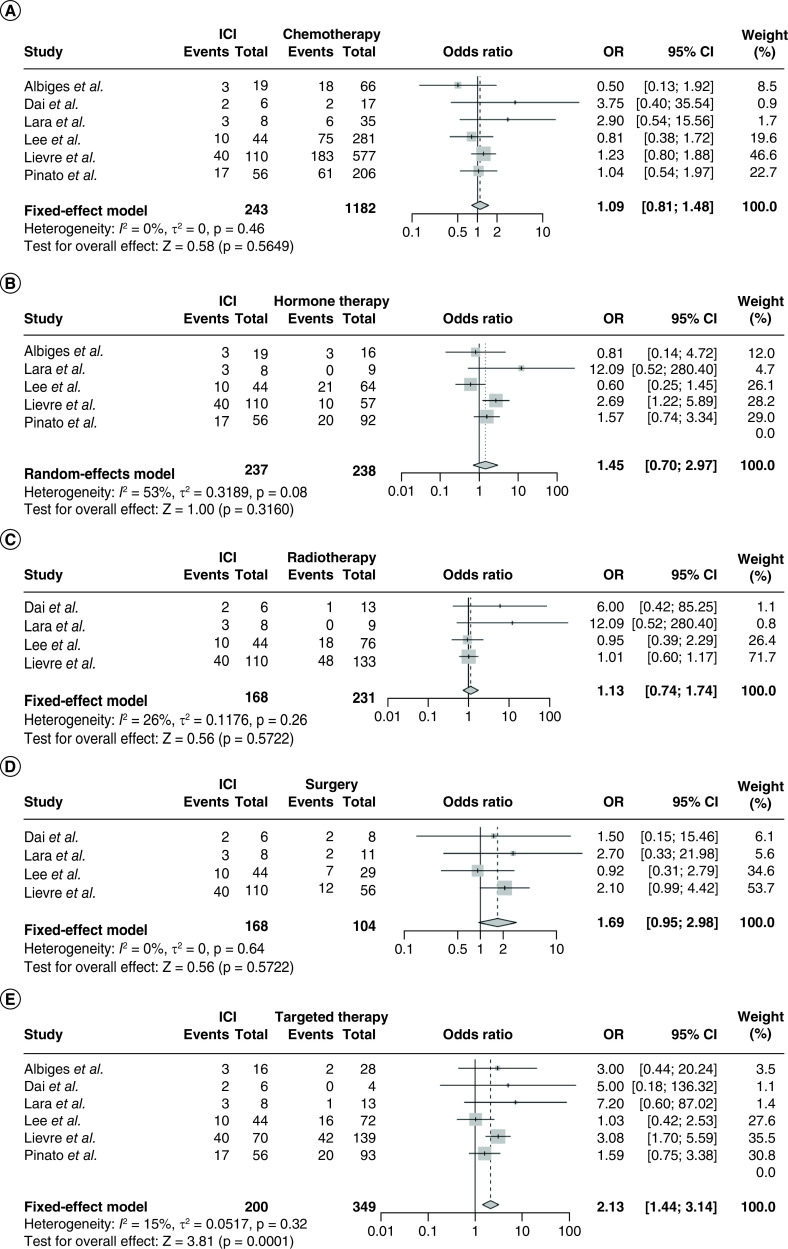
The pooled mortality of COVID-19 infection. The mortality was compared between patients with prior ICI treatment and those with **(A)** chemotherapy; p = 0.56, **(B)** hormone therapy; p = 0.32, **(C)** radiotherapy; p = 0.57, **(D)** surgery; p = 0.57 or **(E)** targeted therapy; p < 0.01. ICI: Immune checkpoint inhibitor; OR: Odds ratio.

We further examined the mortality between exposure to ICI and other treatments in cancer patients in the context of COVID-19. However, we did not identify significant differences between ICI and chemotherapy (OR 1.09, 95% CI 0.81–1.48, p = 0.56; I^2^ = 0%; Figure 3A), hormone therapy (OR 1.45, 95% CI 0.70–2.97, p = 0.32; I^2^ = 53%; Figure 3B), radiotherapy (OR 1.13, 95% CI 0.74–1.74, p = 0.57; I^2^ = 26%; Figure 3C), surgery (OR 1.69, 95% CI 0.95–2.98, p = 0.57; I^2^ = 0%; Figure 3D), except for targeted therapy (OR 2.13, 95% CI 1.44–3.14, p < 0.01; I^2^ = 15%; Figure 3E).

### Temporal relationship between prior ICI receipt & diagnosis of COVID-19

**Figure 4.**
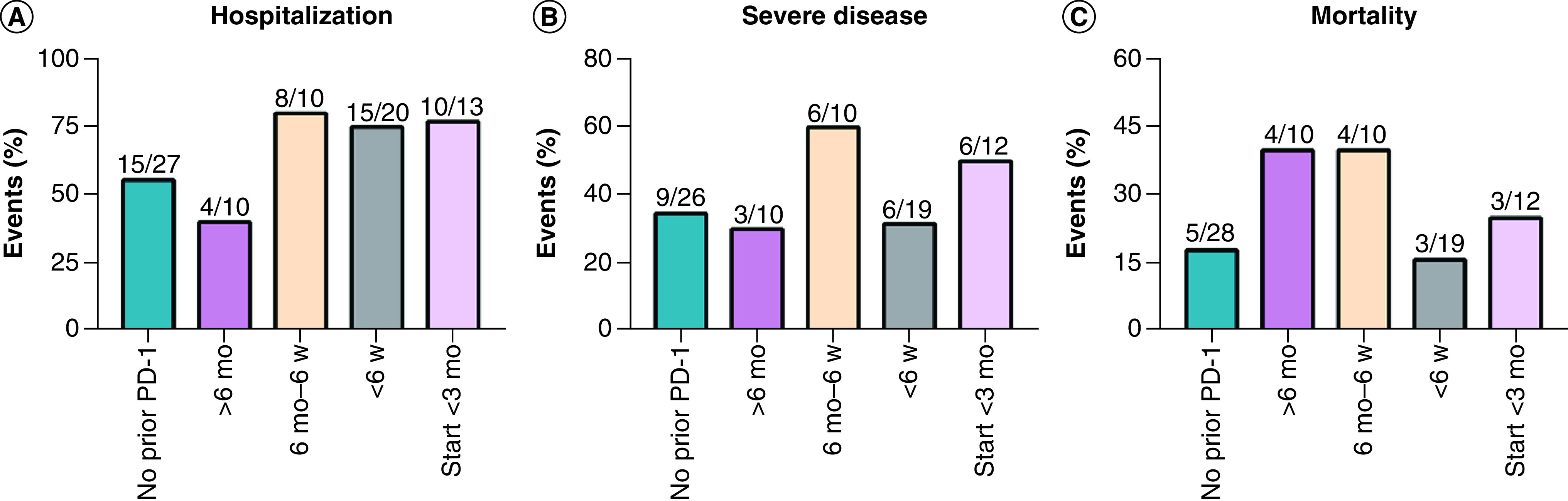
The impact of prior PD-1 exposure on the prognosis of COVID-19 in patients with lung cancer. Patients were redistributed into five groups: no prior PD-1, interval >6 months (>6 mo), interval between 6 months and 6 weeks (6 mo–6 w), interval <6 weeks (<6 w) and initial dose within 3 months (start <3 mo). **(A)** Rate of hospitalization compared between no prior PD-1 and >6 mo (p = 0.48), 6 mo–6 w (p = 026), <6 w (p = 0.17) and start <3 mo (p = 0.30). **(B)** Rate of severe disease compared between no prior PD-1 and >6 mo (p = 1.00), 6 mo–6 w (p = 0.26), <6 w (p = 0.38) and start <3 mo (p = 0.48). **(C)** Rate of death compared between no prior PD-1 and >6 mo (p = 0.24), 6 mo–6 w (p = 0.24), <6 w (p = 1.00) and start <3 mo (p = 0.68). mo: Month; w: Week. Data taken from [23].

Given that the receptor can be occupied for months [30] and the initial start of ICI therapy results in a distinct proliferative burst [31–34], different intervals from the last dose of ICI to the diagnosis of COVID-19 may theoretically influence the prognosis of COVID-19 infection. Luo *et al.* [23] defined five categories of prior PD-1 blockade, including no prior PD-1, ever received PD-1 blockade, last receipt within 6 months, last receipt within 6 weeks, and first receipt within 3 months, detecting the outcomes of interest. Overall, there was no significant difference in prognosis regardless of PD-1 blockade exposure. We extracted data from this study and regrouped patients according to intervals from last dose of ICI to the diagnosis of COVID-19: no prior PD-1, interval >6 months, interval between 6 months and 6 weeks, interval <6 weeks and initial dose within 3 months (Figure 4). However, we did not capture any statistically significant differences between no prior PD-1 group and the other four groups tested by chi-square test or Fisher’s exact test in terms of prognosis, including hospitalization, severe disease and mortality (Figure 4). Consistent with the above outcomes, Wu *et al.* [29] observed a similar risk of severity in different intervals from the last ICI administration to COVID-19 diagnosis (interval ≥28 days vs interval <28 days, p = 1.00).

### ICI-induced lung injury & COVID-19 infection

ICI-induced pneumonitis presents similar clinical and radiological features to COVID-19, challenging the early diagnosis of COVID-19 [35]. Guerini *et al.* [36] and Lovly *et al.* [37] reported two cases where patients experienced misdiagnosis caused by ICI-induced pneumonitis and later died due to an uncontrolled COVID-19 infection. Clinicians should always consider COVID-19 as a differential diagnosis, as few places were spared during the pandemic. In another report [38], two patients were initially highly suspected of COVID-19 infection based on clinical manifestations, imaging findings and epidemiology. Steroids were withheld in one case, and the disease became worse until a third CT scan was obtained, and a second negative RT-PCR test was released after admission. Both patients were eventually diagnosed with ICI-induced pneumonitis, and a mean delay of 3 days in steroid initiation was attributed to the COVID-19 pandemic.

Except for missed window of optimal treatment caused by delayed diagnosis, ICI-induced pneumonitis itself reduces patient resistance and exacerbates COVID-19 infection. Here, we were curious about the influence of ICI in lung cancer patients infected with COVID-19. Data showed that ICI application did not significantly influence the severity of COVID-19 in lung cancer patients (ICI application [7/12] vs no ICI application [8/23], p = 0.181) [27]. Consistently, ICI exposure in lung cancer patients did not exhibit a higher risk for developing severity than in patients with other solid cancers (lung cancer [7/12] vs other solid cancers [5/19], p = 0.13) [27].

### Publication bias

The results of publication bias was shown in Table 2, which was assessed using the Begg's funnel plot and Egger's test. There was no significant publication bias in the included studies (all p > 0.5).

## Discussion

This review included 13 articles that encompassed 409 ICI users infected with COVID-19. It is uncertain whether prior exposure to ICI increases the risk of severe disease and death but, observed OR suggests a higher rate of hospitalization. In addition, different intervals from the last dose of ICI to diagnosis of COVID-19 might not influence the prognosis of COVID-19 infection. Finally, given the unpredictable duration of the pandemic, we should always keep in mind a differential diagnosis of COVID-19 and rational adjustment of ICI use.

Patients with cancer are theoretically more vulnerable to infection due to poor health status and immunosuppressive conditions provoked by both the cancer and antitumor therapies [39–42]. Poorer prognosis in COVID-19 infection has been associated with several factors, including older age, gender and comorbidities such as pulmonary disease, cardiac disease, hypertension and cancer [26,43]. Liang *et al.* collected and analyzed 1590 cases from 575 hospitals [7]. In their study, 18 of 1590 (1%; 95% CI 0.61–1.65) COVID-19 cases had a history of cancer, which was higher than the overall incidence of cancer in the population (285.83 [0.29%] per 100,000 people). Importantly, patients with cancer exhibited a higher rate of severe disease than patients without cancer (7/18 [39%] vs 124/1572 [8%], p = 0.0003). Here, we pooled the prognostic data from COVID-19-infected cases with prior exposure to ICI. The rate of hospitalization was 45%, 34% developed severe disease and 26% died.

Physicians worry about the influence of ICI administration on COVID-19 infection for two main reasons [44]. The first is the potential overlap between the two lung injuries: possible pneumological toxicity from ICI use and COVID-19 pneumonia. The incidence of ICI-related pneumonitis was reported to be 2.5–5% with anti-PD-1/PD-L1 monotherapy and 7–10% with anti-CTLA-4/anti-PD-1 combination therapy [45]. These fatal immune-related adverse events accounted for 35% of treatment-related deaths [46]. The second concern is the potential synergy between ICI mechanisms and COVID-19 pathogenesis, both of which are involved in immune hyperactivation [47–49]. Integrating multiple studies into the present study, we found that prior receipt of ICI significantly increased the rate of hospitalization. In contrast, there was no significant difference in severe disease and mortality among patients with or without prior ICI exposure (OR 1.55, 95% CI 0.69–3.51, p = 0.29; OR 1.12, 95% CI 0.85–1.48, p = 0.42; Figure 2B & C). We speculate that prior ICI exposure may lead to gastrointestinal and respiratory symptoms in some patients, which could contribute to more hospitalization.

Most of the included studies focused on the impact of ICI use or not on the prognosis of COVID-19 [8,18–22], but they did not take other important factors into consideration, including courses of ICI use, intervals from the last dose to the diagnosis of COVID-19, and the effect of the first dose. Wu *et al.* [29] found that patients who received three or more cycles of ICI were more likely to develop severe COVID-19, albeit this difference was not statistically significant (6/7 [85.7%] vs 1/4 [25%], p = 0.09). Another study [23] included 69 patients and defined five groups according to the interval from last ICI receipt to COVID-19 diagnosis. Overall, there was no statistically significant difference in different groups in terms of the rate of hospitalization, severe disease or death.

As the influence of ICI on cancer patients infected with COVID-19 is not clear, there are no authoritative guidelines for ICI modifications in the context of COVID-19. Modifications of drug application are often empirical and based on the mechanism of drug action, taking tumor treatment and epidemic prevention into account [50]. Wang [51] *et al.* suggested that administration of anticancer drugs should be changed from infusion to oral administration if available. For maintenance therapy, we could appropriately prolong the infusion intervals according to patient condition. Aeppli *et al.* performed an online survey among physicians involved in the treatment of renal cell carcinoma [14]. Compared with that outside the pandemic, the use of ipilimumab/nivolumab fell by half in intermediate/poor-risk patients during the pandemic (80 vs 41%). In patients responding to established ICI-containing therapies, most participants modified treatment regimen by extending cycle length. Another survey focused on patient perspective on oncological care [52]. In patients with adjusted treatment, immunotherapy (32%) was most frequently adjusted. Consistently, in patients with delay and discontinuation of treatment (39 and 33%, respectively), immunotherapy was the most frequently included modality.

This study has important implications for clinical practice. Given that the pandemic may last for another several months or even years, physicians should balance cancer treatment and COVID-19 infection. Our results indicate that ICI administration increases the rate of hospitalization, though it is uncertain whether prior exposure to ICI increases the risk of severe disease and death. This suggests that we should not easily postpone, suspend or alter our established treatment decisions in clinical practice, especially for patients who are undergoing ICI-containing regimens, because ICI has irreplaceable performance in certain antitumor treatments [12]. Delay or modification of therapy should be considered on a case-by-case basis [53].

This systematic review and meta-analysis has several limitations. The most important limitation is that we could not rule out unknown confounders. Previous studies reported that age, sex, smoking and comorbidities, including pulmonary disease, cardiac disease, and hypertension, significantly affect the prognosis of COVID-19 infection. However, these potential confounders were not considered in most of the included studies. What is more, in the absence of a head-to-head comparison between ICIs, the choice to perform an evaluation as a new pharmacological class is theoretically unsound. However, among all the researches enrolled in the binary outcome, we found that none of the studies could provide prognosis information about the different ICI molecules. Second, due to the relatively small number of studies, we were unable to evaluate the effects of ICI subclasses or line of treatment or their role in individual tumors. Low proportion of patients treated with immunotherapy would unavoidably confound the meta-analysis results to some extent. Additionally, the benefit of longer duration of ICI on the overall survival has been determined, and frequent or early interruption of ICI has been proved to be associated with worse overall survival [12]. It will be worthwhile to comment on the cancer outcome in this population. However, limited by a short follow-up period, we failed to assess the cancer outcome in patients who had delayed or interrupted ICI treatment through published studies. Further, studies on the association between immune-related adverse events and COVID-19 risk, and outcome are needed. At last, included studies defined several intervals from the last dose to the diagnosis of COVID-19 infection, which may have influenced the findings.

## Conclusion

The results of this meta-analysis suggest that a higher rate of hospitalization was observed among patients who were undergoing ICI-containing regimens, although it is uncertain whether prior exposure to ICI increases the risk of severe disease and death. Additionally, different intervals from last dose of ICI to the diagnosis of COVID-19 may not influence the prognosis of COVID-19 infection.

**Summary points**

- The influence of prior exposure to immune checkpoint inhibitors (ICIs) on COVID-19 infection remains largely unknown.
- It is necessary to perform a meta-analysis to quantify the effects of ICI on the prognosis of COVID-19.
- We systematically searched the PubMed, Embase and Web of Science databases.
- We included studies that reported ICI use in cancer patients and their prognosis in the context of COVID-19.
- Chi-squared and I^2^ tests were used to calculate heterogeneity among the included studies, and the choice of random or fixed effects model was made according to the heterogeneity.
- Thirteen studies comprising 4614 cancer patients with COVID-19 were included for the systematic review and meta-analysis.
- The pooled rate of hospitalization, severe disease, and mortality in patients with prior exposure to ICI was 0.45 (95% CI 0.15–0.78), 0.34 (95% CI 0.26–0.44) and 0.26 (95% CI 0.17–0.38), respectively.
- When compared with cancer patients without prior ICI exposure, patients with prior ICI treatment exhibited a higher rate of hospitalization (odds ratio 2.0, 95% CI 1.19–3.38, p = 0.01).
- No statistically significant difference in mortality was observed between patients exposed to ICI and other antitumor treatments in the context of COVID-19, except for the targeted therapy.
- It is uncertain whether prior exposure to ICI increases the risk of severe disease and death but observed odds ratio suggest a higher rate of hospitalization.
